# A Four-Year Survey of Hemoparasites from Nocturnal Raptors (Strigiformes) Confirms a Relation between *Leucocytozoon* and Low Hematocrit and Body Condition Scores of Parasitized Birds

**DOI:** 10.3390/vetsci10010054

**Published:** 2023-01-12

**Authors:** Bárbara Martín-Maldonado, Aida Mencía-Gutiérrez, Cristina Andreu-Vázquez, Rocío Fernández, Natalia Pastor-Tiburón, Alberto Alvarado, Alicia Carrero, Aitor Fernández-Novo, Fernando Esperón, Fernando González

**Affiliations:** 1 Department of Veterinary Medicine, Faculty of Biomedical and Health Sciences, Universidad Europea de Madrid, 28670 Villaviciosa de Odón, Spain; 2 Wildlife Hospital, Grupo de Rehabilitación de la Fauna Autóctona y su Hábitat (GREFA), 28220 Majadahonda, Spain; 3 Department of Medicine, Faculty of Biomedical and Health Sciences, Universidad Europea de Madrid, 28670 Villaviciosa de Odón, Spain; 4 Departmental Section of Pharmacology and Toxicology, Faculty of Veterinary Science, University Complutense of Madrid, 28040 Madrid, Spain

**Keywords:** blood parasites, Strigiformes, wild birds, *Leucocytozoon*, *Haemoproteus*

## Abstract

**Simple Summary:**

Parasitism is one of the most common life strategies on Earth, where the host and the parasite establish a successful relationship and continually adapt to each other. Most of the studies on wild birds show that those with hemoparasites are usually asymptomatic due to this host–parasite coevolution, so blood parasites are often detected as incidental laboratory findings. Most of these studies have been performed mainly in passerines and migratory species, but nocturnal raptors seem to be more exposed to blood parasite vectors than other avian species due to their behavior and distribution. Blood samples were collected from 134 individuals during a four-year period to assess the occurrence of blood parasites and parasitemia in different species of nocturnal raptors and their effect on hematological parameters. Thirty-five percent (95% CI: 27.5–43.5%) of individuals included in the study tested positive for at least one hemoparasite genus, and 11.2% showed coinfection. *Leucocytozoon* was the genus most frequently detected (32.1%), followed by *Haemoproteus* (11.2%), *Trypanosoma* and *Plasmodium* (2.2% each). The Eurasian eagle-owl *(Bubo bubo*) was the species with the highest prevalence (94.7%). Moderate anemia and an increase in leukocyte counts were detected in the positive birds. Moreover, the positive animals showed a poor body condition score.

**Abstract:**

Most hemoparasites hosted by wild birds appear to be harmless, but most of the blood parasite studies in avian wildlife are mainly focused on passerines or migratory species. This study aimed to assess the occurrence of blood parasites in nocturnal raptors (Strigiformes order) and their effect on hematological parameters. A total of 134 blood samples were collected during a four-year period for hematological analysis and hemoparasite detection and quantification by microscopical examination of the samples. Overall, the occurrence of hemoparasites was 35.1%, with *Leucocytozoon* being the most frequently detected (32.1%), followed by *Haemoproteus* (11.2%), *Trypanosoma* and *Plasmodium* (2.2% each). Among the different bird species, the Eurasian eagle-owl *(Bubo bubo*) showed the highest blood parasite positivity (94.7%). In barn owls, the positive birds displayed a lower hematocrit measurement and body condition score than the non-parasitized ones (*p* = 0.007 and *p* = 0.005, respectively), especially those parasitized by *Leucocytozoon*. Moreover, the analysis of the magnitude of this association revealed that the presence of hemoparasites is five times more frequent in barn owls with a 2/5 body condition score. Despite the host–parasite coevolution in Strigiformes, our results show a correlation between the presence of hemoparasites and some health parameters, including blood parameters.

## 1. Introduction

Parasitism is one of the most common life strategies on Earth. The host–parasite relationship is the result of successful coevolution where both continually adapt to each other [1], and most parasites can infect a large variety of species without causing disease [2]. In this sense, the majority of hemoparasites occurring in birds appear to be harmless to the host, especially for strongly host-specific genera such as *Haemoproteus* and *Leucocytozoon* [3]. Although most of the cases of these two genera are detected as incidental laboratory findings, clinical manifestations may develop in animals with physiologically challenging states, chronic stress conditions, or debilitating synergistic diseases [4,5,6,7]. Naïve host species also seem to be more susceptible to clinical levels of blood parasite infection [7]. Clinical signs and severity vary widely among individuals, including weight loss, anemia, air sacculitits, arthritis, seizures, or even reduced lifespan and death [8,9].

Most of the blood parasite studies in wild birds have been conducted on Passeriforme species due to the easy capture of these individuals or on migratory bird species, as migration can enhance the parasite transmission to different or novel locations [10,11,12,13]. Only a few studies have been conducted on non-migratory species—who could play the role of sentinels of the environmental health status of a specific region [7,8,9,14]. 

Birds of prey are at the top of the food chain and play an important role in maintaining ecological balance [15]. For that reason, during the past few years, some studies have been published focusing on raptors [9,16,17,18,19]. A thirteen-year study in Beijing revealed that 18% of diurnal raptors tested (Accipitriformes and Falconiformes orders) were positive for blood parasites, in contrast to 38% of nocturnal raptors [19]. While most of the studies conducted on birds of prey suggest the asymptomatic nature of blood parasite infection, some authors have confirmed the involvement of blood parasites in the death of snowy owls (*Bubo scandiacus*) [9,16,17,20]. 

Currently, there are more than 250 different species of blood parasites described in birds [9], with *Leucocytozoon* being the most frequent genera, followed by *Haemoproteus*, *Plasmodium* and *Trypanosoma* [7,19,21]. Gao et al. (2021) detected that *Haemoproteus* and *Leucocytozoon* presence was significantly higher in nocturnal raptors than in diurnal raptors while there were no differences for *Plasmodium*. All of these are vector-borne parasites globally dispersed [7]. 

Vector distribution is one of the key factors for blood parasite occurrence in birds. Ambient humidity and temperature are two factors that determine the development of vectors and parasites [22]. The Iberian Peninsula is a Mediterranean region with a temperate climate and a clear seasonality that favor the development of hemoparasite vectors [23]. In addition, global warming and climate change may affect the traditional distribution of vectors who move to new regions with naïve host species [7]. Moreover, Spain has been described as a biodiversity hotspot, especially for birds, and a suitable region to stop during migration, which can increase the transmission of parasites between individuals and even different bird species [24,25]. 

In this context, the present study aimed to assess the prevalence of different species of blood parasites in nocturnal raptors from central Spain and their effect on hematological parameters.

## 2. Materials and Methods

### 2.1. Study Population and Sample Collection

All samples were collected from nocturnal raptors of central Spain between 2018 January and 2021 December, from individuals admitted to GREFA (Grupo de Rehabilitación para la Fauna Autóctona y su Hábitat) wildlife hospital or free-living birds handled during the banding process, prior to any treatment or housing in the hospital cages. Bird-handling procedures were performed for clinical purposes and according to Directive 2010/63/EU on the protection of animals used for scientific purposes [26].

Information about sex, age, body condition score and season of sampling was recorded to study the potential relationship between those parameters and the presence and quantification of blood parasites. The age was estimated by the feather development, and then the animals were grouped into young (nestlings and fledglings) or adult clusters based on this assessment. Sex was determined, when possible, by molecular analysis at the Laboratorio Central de Veterinaria (Algete, Spain) [27]. Finally, the body condition score (BCS) of each animal was estimated by morphometry using a one-to-five system where level 1 was for emaciated, level 2 for under-conditioned, level 3 for well-conditioned, level 4 for over-conditioned, and level 5 for obese individuals [28].

For the hematological analysis and parasite detection, brachial or jugular venipuncture was performed with a 1 mL syringe and a 25-gauge needle to obtain a 250 μL blood sample. Immediately, two thin blood films were smeared on clean glass slides with a small drop of fresh blood (20 μL) and air dried. The rest of the blood sample was preserved with EDTA (ethylenediaminetetraacetic acid) with calcium and stored at 4 °C.

### 2.2. Laboratory Analysis

The hematological analysis was performed within 24 h after collection, including two different tests: hematocrit measurements and a blood smear. Regarding hematocrit measurement, a hematocrit capillary was filled with the preserved blood and centrifugated at 11,000 rpm for 3 min. The proportion of erythrocytes was calculated and registered [29]. For the blood smear, the blood extension performed during sample collection with fresh blood was fixed with pure methanol after drying and stained with the modified Wright–Giemsa stain, following the Samour protocol, and then observed under a 100× microscope lens [29]. One hundred leukocytes were classified as heterophiles, lymphocytes, eosinophiles, basophiles or monocytes, and an H/L ratio was calculated. White blood cell (WBC) estimation was obtained by counting leukocytes from ten different fields, under the 40× Motic BA210E light microscope lens (Motic^®^, Schertz, TX, USA). The mean of leukocytes per field was multiplied by 2000 to obtain an estimated WBC count according to Fudge (2000) [30]. 

Then, hemoparasite detection was performed by the gold standard method. Briefly, each smear was examined for 30 min, which is equivalent to approximately 150 fields, by a single expert observer. When no parasites were detected, the sample was considered negative. Larger parasites, including *Leucocytozoon* and *Trypanosoma*, were detected at 400×. For smaller parasites, oil immersion microscopy (1000×) was needed. Because the speciation of blood parasites could be too complex based only on morphology, the parasite identification was made just to the genus level according to the descriptions of Valkiūnas (2005) and Tostes et al. (2017) [7,31].

Quantification was estimated in homogeneous monolayer fields. Twenty random fields were analyzed for a total of approximately 5000 RBC (red blood cells). Within each field, the numbers for each hemoparasite species were recorded. The level of parasitemia for each parasite genus was then calculated and reported per 1000 RBC [20]. Finally, infection intensity was clustered into three categories: low (1–5 parasites/1000 RBC), medium (5–10 parasites/1000 RBC) or high intensity (>10 parasites/1000 RBC).

### 2.3. Statistical Analysis

Normal distribution of data was assessed with the Shapiro–Wilk test, and continuous variables are expressed as mean and SD or as median and interquartile range [Q1, Q3], as appropriate. Categorical variables are expressed as absolute and relative frequencies (n and percentage). For each species and hemoparasite genus, the proportion of parasitized animals and its 95% confidence interval (CI) were calculated. 

In barn owls (*Tyto alba*), Chi-squared test and Fisher’s exact test were used to identify potential factors associated with the presence of hemoparasites (sex, age group, season of sampling, and year of sampling). Univariate and (when appropriate) multivariate logistic regression analysis were used to calculate the odds ratio and its 95% CI for statistically significant variables. Differences in the hematological parameters and the heterophiles/lymphocytes (H/L) ratio between parasitized and non-parasitized barn owls were assessed with an independent Student’s *t*-test or a Mann–Whitney U test, according to data distribution. These analyses could not be performed on the other species because of the sample size.

In the Eurasian eagle-owls (*Bubo bubo*), Kruskal–Wallis tests were used to assess the differences in the hematological parameters among animals with low (1–5 parasites/1000 RBC), medium (5–10 parasites/1000 RBC) and high (>10 parasites/1000 RBC) parasitemia levels of each parasite genus. These analyses could not be performed in the other species due to sample size.

Differences at the level of *p* < 0.05 were considered statistically significant. Statistical analyses were performed using the STATA BE 17.0 software package (StataCorp LLC, Aurora, IL, USA).

## 3. Results

### 3.1. Study Population

A total of 134 individuals from six species of nocturnal raptors were included in the study: 90 barn owls (Tyto alba), 19 Eurasian eagle-owls (Bubo bubo), 10 tawny owls (Strix aluco), 10 European owls (Athene noctua), 4 Eurasian Scops-owls (Otus scops) and 1 Northern long-eared owl (Asio otus). Molecular analysis for *sex* determination was possible in only 58 of the 134 animals (77.7%). Details of population distribution are summarized in Supplementary Table S1.

### 3.2. Hemoparasite Detection and Quantification

**Figure 1:**
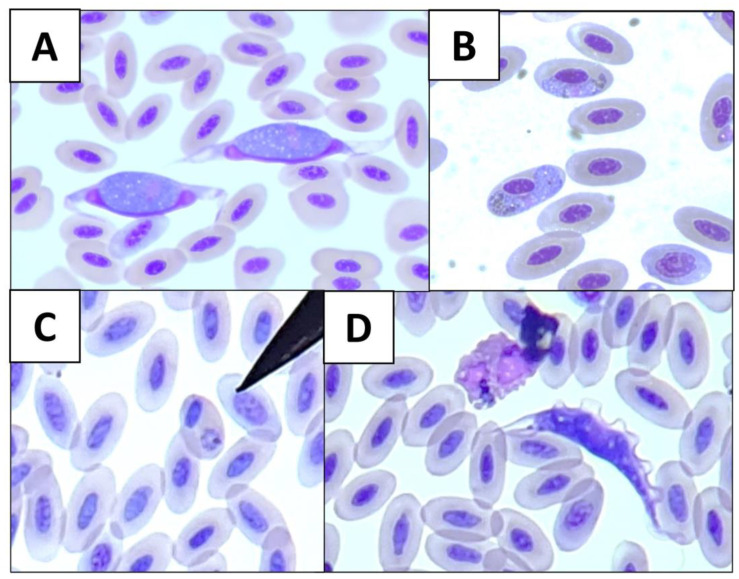
Microscopical photographs of blood parasite genera from wild birds at 1000×: (**A**) *Leucocytozoon*; (**B**) *Haemoproteus*; (**C**) *Plasmodium*; and (**D**) *Trypanosoma*.

From the animals tested in this study, 35.1% (47/134) were positive for at least one blood parasite genus. Microscopic examination of blood smears revealed four morphologically distinguishable genera of blood parasites (Table 1). *Leucocytozoon* was the genus most detected (43/134; 32.1%), followed by *Haemoproteus* (15/134; 11.2%), *Trypanosoma* and *Plasmodium* (3/134; 2.2% each) (Figure 1). Regarding the host species, the Eurasian eagle-owl was the nocturnal raptor with highest blood parasite rate (18/19; 94.7%). Moreover, the Eurasian eagle-owl and tawny owl were the only raptor species in which the four blood parasite genera have been observed. The only Northern long-eared owl included in the study was positive for *Haemoproteus*. 

**Figure 2:**
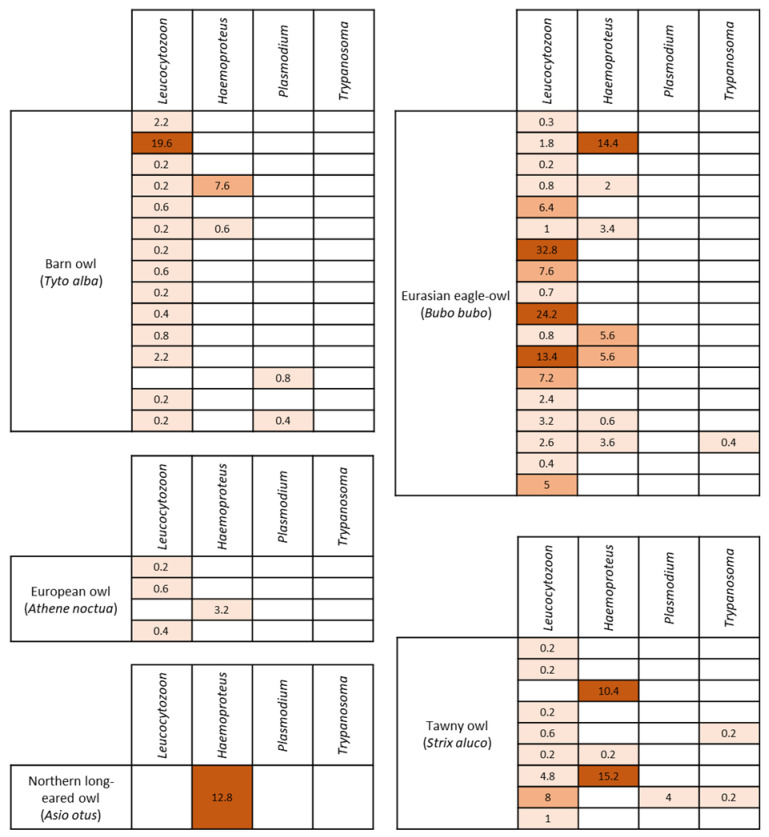
Detection of the four different genera of hemoparasites according to bird species and heat map of parasitemia (expressed in parasites per 1000 RBC).Finally, coinfection was observed in 31.9% of parasitized animals (15/47). The Eurasian eagle-owl was the species with the highest occurrence of coinfection (8/19; 42.1%), followed by tawny (4/10; 40%) and barn owls (3/90; 3.3%). The *Leucocytozoon–Haemoproteus* combination was the most detected. Only two individuals carried three different genera: a Eurasian eagle-owl with a *Leucocytozoon*, *Haemoproteus* and *Trypanosoma* coinfection, and a tawny owl with a *Leucocytozoon*, *Plasmodium* and *Trypanosoma* coinfection. Indeed, *Trypanosoma* was the only parasite detected in combination with another blood parasite genus. No coinfection was detected in European owls, Eurasian Scops-owls, or Northern long-eared owls.

Overall, the parasitemia level of hemoparasites was low (0.1–5 parasites/1000 RBC), but the birds positive for *Haemoproteus* showed the highest load, especially in the tawny owl (10.4 [0.2, 15.2] parasites/1000 RBC [Q1, Q3]) and the Northern long-eared owl (12.8 [12.8, 12.8] parasites/1000 RBC). In contrast, the birds parasitized by *Trypanosoma* showed the lowest parasitemia level (0.2 [0.2, 0.2] parasites/1000 RBC) (Table 1). Regarding the bird species, the Eurasian eagle-owl had the highest occurrence of blood parasites, and the parasitemia level of each parasite genus was low. The infection intensity of each positive animal is detailed in Figure 2. In the Eurasian eagle-owl, no association among the parasitemia of each parasite genus and the hematological parameters was detected.

### 3.3. Analysis of Epidemiological Variables

Looking at the distribution of blood parasites among the study years, 2018 was the year with the highest occurrence of blood parasites: 17 positive individuals from a total of 36 tested birds (47.2%). In contrast, in 2019 only 7 individuals carried blood parasites (7/34; 20.6%). *Leucocytozoon* stands out as the most detected genus among the years. However, *Plasmodium* was detected only in 2021. No statistical differences have been detected among different years (*p* = 0.139). 

However, according to the season, a statistical trend has been detected in barn owls and tawny owls. In barn owls, positivity rate was different among sampling seasons (*p* = 0.064), reaching 22.9% (14/61) in summer, 5% (1/20) in spring and 0% (0/9) in autumn. No barn owls were sampled in winter. The same relation has been detected in tawny owls, but the number of individuals is too small to be considered a statistical difference (*p* = 0.019). *Leucocytozoon* and *Haemoproteus* were observed in all the seasons, while *Plasmodium* was detected only in spring and summer, coinciding with the rising temperature and vector population. The four blood parasite genera were detected in spring and summer, but not in autumn or winter. 

The comparison of BCS values between parasitized and non-parasitized birds manifested a statistical difference in barn owls: the presence of hemoparasites was more frequent in individuals with a 2/5 body condition score than in individuals with a 3/5 or higher body condition score (*p* = 0.005). Moreover, the analysis of the magnitude of this association revealed that the presence of hemoparasites is five times more likely in barn owls with a 2/5 body composition score compared with barn owls with a 3/5 body condition score (Odds ratio = 5.0; 95% CI: 1.5–16.6). 

Regarding the age of the animals, *Trypanosoma* was only detected in adult raptors, while the other genera were detected in both young and adult individuals. However, no statistical differences have been observed according to the age or sex of animals.

### 3.4. Results of Hematological Parameters

Results of the hematological analysis were slightly different between different owl species. While the hematocrit values of owls are quite similar, the WBC estimated count varies from 10.250 cells/mm^3^ in tawny owls to 28.375 cells/mm^3^ in Eurasian Scops-owls. The leukocyte formula also remains similar between owl species, with a clear predominance of heterophils in all six species. Basophil cells had the lowest count. Details of the hematological values in the different bird species are summarized in Table 2. 

Of all the barn owls, six individuals did not have a quality sample in order to perform a complete hematological analysis. Statistical differences were detected on the hematocrit measurement and the WBC count (Table 3). 

In the parasitized barn owls, the hematocrit measurement results were significantly lower (35%; [30%, 39%]) than in the non-parasitized individuals (39%; [35%, 43%]) (*p* = 0.007). Moreover, a statistical association has been detected between the presence of *Leucocytozoon* and the hematocrit measurements of infected barn owls, where birds infected by *Leucocytozoon* showed lower hematocrit values (*p* = 0.018). On the contrary, the presence of *Haemoproteus* did not infer in the hematocrit value (*p* = 0.967).

The WBC count was significantly higher in parasitized barn owls (35,275 [20,750, 40,200] WBC/mm^3^) than in non-parasitized birds (20,500 [11,625, 26,875] WBC/mm^3^) (*p* = 0.003). Moreover, a trend has been detected on the ratio of H/L, which was higher in parasitized birds than in non-parasitized ones (*p* = 0.054). If the H/L ratio was evaluated according to each parasite species, this trend was observed only in birds infected by *Leucocytozoon* (*p* = 0.073). Detailed results of the comparative analysis of hematological values between both groups of barn owls are described in Table 3. 

## 4. Discussion

The present study is a four-year retrospective survey of blood parasite presence in wild nocturnal raptors from Spain. Overall, the occurrence of hemoparasites was 35.1%, which is in concordance with previous reports in the Iberian Peninsula but higher than other studies carried in France and Germany [15,17,23,32,33,34]. However, some authors had obtained a positivity of up to 73.6% of Strigiformes parasitized in Eastern Europe, 73.1% in North America, and 72.2% in South America [4,21,35,36,37].

In our study, the presence of blood parasites seems to vary among bird species. The Eurasian eagle-owl and tawny owl are the species with higher percentage of parasitized individuals with a 94.7% and 90% positivity, respectively. Similar results had been obtained in these species previously [9,32,34,37]. Moreover, species from the genus *Bubo* seem to have a great affinity to some genera of hemoparasites, namely *Leucocytozoon* and *Haemoproteus*; however, the co-evolution of species has favored an immunological adaptation [38]. The lowest prevalence of blood parasites was detected in barn owls, which agrees with the findings of Ishak et al. [4].

Among the different genera of hemoparasites detected in nocturnal raptors, the most frequent in the scientific literature remain *Leucocytozoon*, *Haemoproteus*, and *Plasmodium* in decreasing order. Although 91.5% of positive animals hosted *Leucocytozoon* (43 of the 47 parasitized birds), *Haemoproteus* was the parasite with the highest parasitemia levels (up to 12.8 parasites/1000 RBC in the only Northern long-eared owl included in the study). *Haemoproteus* and *Leucocytozoon* are relatively common in wild birds. In fact, thanks to the co-evolution and adaptation of the host immune system, blood parasites are considered benign in healthy nocturnal raptors, which maintain a low parasitemia. However, the susceptibility to hemoparasites can vary if the host is dealing with other pathologies or stress. These factors can reactivate the infection, thereby increasing the parasitemia. In this sense, parasitemia can be considered as a good indicator of the health status of the Strigiformes species [20,39]. 

On the contrary, for *Trypanosoma*, the present study shows a low occurrence and a low level of parasitemia. Since trypanosomiasis is considered a tropical disease, and most of the clinical cases reported in Europe have been linked to globalization and travels, there are only a few *Trypanosoma* species occurring naturally in European wildlife, including *T. lewisi*, *T. melophagium* or *T. theileri* [40]. In this context, studies about trypanosome species identification are scarce; however, some authors have published the detection of *T. corvi*, *T. bennetti* and *T. avium* in wild birds from Central and Northern Europe [12,41,42].

It is important to highlight that the prevalence of blood parasites in wildlife depends directly on the exposure of those birds to vectors. Increased blood parasite infection has been associated with an increase in the presence of vectors, related to ecological niches, geographical region, season, migration, and environmental factors such as temperature or humidity [4,33]. In this sense, Mediterranean areas do seem to obtain a higher percentage of blood parasites in late spring, summer, and early autumn, when climatic conditions are better for the development of vector populations. In fact, our results show a trend in barn owls and tawny owls (*p* = 0.064 and *p* = 0.019, respectively), with a higher occurrence of infection in summer. Baker et al. (2018) suggested that heat stress could reactivate chronic infections into acute infections, which explains the highest detection in this season [20]. Some authors have also related breeding and migration with the highest incidences because the demand for high energy coupled with a lower body condition can lead to a decrease in immunity in those species [19]. Moreover, migration can increase exposure to biting vectors for transmission of blood parasites to avian hosts [4,21]. Finally, some authors suggest that a few *Haemoproteus* lineages can be present and transmitted through all the seasons [36,43,44]. At this point, it is important to remember that the four blood parasite genera detected in the present study were observed in spring and summer but not in autumn or winter. *Trypanosoma* was detected in spring, summer, and winter, suggesting that any parasite species detected in winter may be autochthonous. *Plasmodium* was only detected in spring and summer when temperatures are warmer. 

In recent years, several authors had reported the death of Strigiformes birds due to blood parasites, especially birds from the *Bubo* genus such as the snowy owl (*Bubo scandiacus*) [20,36,38,45,46,47]. Many of the dead Strigiformes birds reported had a poor body condition before the diagnosis [48]. A correlation between the presence of hemoparasites and the body condition score has been established in our study: blood parasites are five times more frequent in barn owls with a 2/5 body condition score. This association has been reported before in the American kestrel (*Falco sparverius*) during breeding season and coinfected house martins (*Delichon urbicum*) [49,50]. Among hemosporidian parasites, *Plasmodium* has been linked to mortality and the decline in population levels of native birds in some regions [18]. Mortality in Strigiformes has been also attributed to coinfections by two or more blood parasite species [4,20,32,35,37,51]. In fact, a coinfection can be a predisposing factor to other pathologies like the West Nile Virus disease, which has been diagnosed in dead parasitized birds [20,52]. This co-occurrence of multiple parasite genera could be due to the adaptation of the immunological system and the co-evolution between the host and the parasite species. The present study shows that coinfections were present in 31.9% of parasitized birds, mainly in the Eurasian eagle-owls and tawny owls. The most frequent combination was *Leucocytozoon–Haemoproteus*, which agrees with data reported by other authors [17,32]. 

On the contrary, immunity to blood parasites may be acquired over the years, so older birds supposedly have more resistance to hemoparasitic effects, and parasitemia levels in those animals should be lower [21,53]. In contrast, young individuals are considered naïve and, therefore, susceptible to blood parasite infection, which can lead to parasite-induced mortality [4,17,21,52]. Nevertheless, our analysis shows that adults had a higher positivity rate than young birds, which could be due to longer cumulative exposure [7,22,46]. 

The hematological effects of hemoparasites, such as anemia, have been previously described in Strigiformes [47,48]. Positive birds from the present study showed a lower hematocrit measurement than non-parasitized ones, especially birds infected by *Leucocytozoon*, which agrees with the authors mentioned above. In mammals, parasites also can induce an increase in the rate of eosinophiles on the leukocyte differential count [54]. However, the function of eosinophiles in birds is still little known. Some previous studies had linked *Leucocytozoon* infections to higher eosinophil counts in raptors [55,56]. Our data did not reveal significant differences on the leukocyte formula between parasitized and non-parasitized birds. In contrast, a trend toward a higher H/L rate in parasitized barn owls has been detected. This result is in accordance with Clark et al. (2016) and Wiegmann et al. (2021), who found increased H/L rates in *Zosterops* spp. infected by *Microfilariae* and raptors infected by hematozoa, respectively [56,57]. In this context, it is important to highlight that the proportion of heterophils and lymphocytes (H/L) is a reliable indicator of immune response to stress or illness in birds, including parasitemia [54].

## 5. Conclusions

Strigiformes from the Iberian Peninsula could be considered hosts of blood parasites from the genera *Leucocytozoon* and *Haemoproteus,* mainly, but also of *Plasmodium* and *Trypanosoma* in a smaller proportion. In barn owls, the presence of hemoparasites, and more specifically *Leucocytozoon*, is related to a lower body condition score. In barn owls, the same parasite genus is associated with a lower hematocrit measurement and a higher H/L rate. These findings bring about the need for further research on the epidemiology and pathology of hemoparasites in nocturnal raptors.

## Figures and Tables

**Table 1:** Prevalence and parasitemia of blood parasite genera detected among the owl species.

Bird Species	Overall Positivity	*Leucocytozoon*		*Haemoproteus*		*Trypanosoma*		*Plasmodium*	
		Prevalence	Parasitemia	Prevalence	Parasitemia	Prevalence	Parasitemia	Prevalence	Parasitemia
Barn owl (*Tyto alba*)	15/90; 16.7% (10.3–25.7%)	14/90; 15.5% (9.5–24.4%)	0.3 [0.2, 2.9]	2/90; 2.2% (0.6–7.7%)	4.1 [0.6, 7.6]	0/90; 0% -	-	2/90; 2.2% (0.6–7.7%)	0.6 [0.4, 0.8]
Eurasian eagle-owl (*Bubo bubo*)	18/19; 94.7% (75.3–99.1%)	18/19; 94.7% (75.3–99.1%)	2.5 [0.8, 6.4]	8/19; 42.1% (23.1–63.7%)	3.6 [2, 5.6]	1/19; 5.3% (0.9–24.6%)	0.4 [0.4, 0.4]	0/19; 0% -	-
Tawny owl (*Strix aluco*)	9/10; 90% (59.6–98.2%)	8/10; 80% (49.0–94.3%)	0.4 [0.2, 2.9]	3/10; 30% (10.7–60.3%)	10.4 [0.2, 15.2]	2/10; 20% (5.6–50.9%)	0.2 [0.2, 0.2]	1/10; 10% (1.8–40.4%)	4 [4, 4]
European owl (*Athene noctua*)	4/10; 40% (16.8–68.7%)	3/10; 30% (10.7–60.3%)	0.5 [0.4, 0.6]	1/10; 10% (1.8–40.4%)	3.2 [3.2, 3.2]	0/10; 0% -	-	0/10; 0% -	-
Eurasian Scops-owl (*Otus scops*)	0/4; 0% -	0/4; 0% -	-	0/4; 0% -	-	0/4; 0% -	-	0/4; 0% -	-
Northern long-eared owl (*Asio otus*)	1/1; 100% (20.6–100%)	0/1; 0% -	-	1/1; 100% (20.6–100%)	12.8 [12.8, 12.8]	0/1; 0% -	-	0/1; 0% -	-
Overall positivity	47/134; 35.1% (27.5–43.5%)	43/134; 32.1% (24.8–40.4%)	-	15/134; 11.2% (6.9–17.6%)	-	3/134; 2.2% (0.7–6.4%)	-	3/134; 2.2% (0.7–6.4%)	-

Data of prevalence are expressed as: n/N—percentage (%) and 95% confident intervals. Data of parasitemia are expressed as: parasitemia average and interquartile range [Q1, Q3] in parasites per 1000 RBC.

**Table 2:** Hematological results according to bird species. Results are expressed with median and quartiles [Q1, Q3].

Blood Parameters	Barn Owl (*Tyto alba*)	Eurasian Eagle-Owl (*Bubo bubo*)	Tawny Owl (*Strix aluco*)	European Owl (*Athene noctua*)	Eurasian Scops-Owl (*Otus scops*)	Northern Long-Eared Owl (*Asio otus*)
	n = 90	n = 19	n = 10	n = 10	n = 4	n = 1
Hematocrit (%)	38 [35, 42]	41 [35, 45]	34 [31, 41]	37 [30, 45]	32.5 [28.5, 39]	39 -
WBC count (cells/mm^3^)	21,000 [13,000, 28,800]	14,935 [9260, 18,995]	10,250 [10,000, 16,750]	14,750 [12,750, 8750]	28,375 [16,960, 36,625]	12,000 -
Heterophils (%)	52 [40, 65]	66 [61, 82]	55 [27, 60]	52 [43, 59]	65 [50–67]	16 -
Eosinophils (%)	4 [0, 10]	2 [0, 5]	3 [0, 5]	1 [0, 2]	1 [0–4]	62 -
Basophils (%)	0 [0, 1]	0 [0, 1]	1 [1, 2]	0 [0, 0]	0 [0, 1]	2 -
Lymphocytes (%)	40 [24, 49]	21 [14, 30]	39 [33, 40]	42 [26, 54]	30 [20–35]	14 -
Monocytes (%)	2 [1, 4]	2 [1, 5]	5 [5, 7]	3 [2, 4]	3 [2–15]	6 -

WBC—white blood cells; %—percentage; n—number of animals. Data are expressed as average value and interquartile range [Q1, Q3].

**Table 3:** Comparison of hematological parameters between parasitized and non-parasitized barn owls using Student’s *t*-test or Mann–Whitney test. Results are expressed with median and quartiles [Q1, Q3].

Blood Parameters	Parasitised n = 15	Non-Parasitised n = 69	*p*-Value
Haematocrit (%)	35 [30, 39]	39 [35, 43]	*p* = 0.007 *
WBC count (cells/mm^3^)	35,275 [20,750, 40,200]	20,500 [11,625, 26,875]	*p* = 0.003 **
Heterophils (%)	61 [41, 76]	50.5 [40, 62]	*p* = 0.124 *
Eosinophils (%)	1 [0, 9]	4 [1, 10]	*p* = 0.216 **
Basophils (%)	0 [0, 1]	0 [0, 1]	*p* = 0.591 **
Lymphocytes (%)	29 [20, 40]	41 [28, 49]	*p* = 0.110 *
Monocytes (%)	3 [0, 5]	2 [1, 4]	*p* = 0.435 **
H/L ratio	2.1 [1.4, 3.8]	1.3 [0.7, 2.2]	*p =* 0.054 **

WBC—white blood cells; %—percentage; H/L—heterophil/lymphocyte ratio; and n—number of animals. Data are expressed as average value and interquartile range [Q1, Q3]. * Student’s *t*-test; ** Mann–Whitney test.

## Data Availability

The data that support the findings of this study are available on request from the corresponding author (B.M.-M.).

## Supplementary Materials

The following supporting information can be downloaded at: https://www.mdpi.com/article/10.3390/vetsci10010054/s1, Table S1: Details about distribution of the study population regarding the species, age, sex and body condition score of animals, and the year of sampling.

## References

[1] Poulin R. (2011). Evolutionary ecology of parasites. Evolutionary Ecology of Parasites.

[2] Palm H.W., Theisen S., Pikalov E., Kleinertz S. (2018). An Update: Manipulation of Fish Phenotype by Parasites. Reference Module in Life Sciences.

[3] Rush E.M., Wernick M., Beaufrère H., Ammersbach M., Vergneau-Grosset C., Stacy N., Pendl H., Wellehan J.F.X., Warren K., LeSouef A., Saunders W.B. (2016). Advances in clinical pathology and diagnostic medicine. Current Therapy in Avian Medicine and Surgery.

[4] Ishak H.D., Dumbacher J.P., Anderson N.L., Keane J.J., Valkiūnas G., Haig S.M., Tell L.A., Sehgal R.N.M. (2008). Blood Parasites in Owls with Conservation Implications for the Spotted Owl (*Strix occidentalis*). PLoS ONE.

[5] Scaglione F., Cannizzo F., Chiappino L., Sereno A., Ripepi M., Salamida S., Manuali E., Bollo E. (2016). *Plasmodium* spp. In a captive raptor collection of a safaripark in northwest Italy. Res. Vet. Sci..

[6] Coker S.M., Hernandez S.M., Kistler W.M., Curry S.E., Welch C.N., Barron H.W., Harsch S., Murray M.H., Yabsley M.J. (2017). Diversity and prevalence of hemoparasites of wading birds in southern Florida, USA. Int. J. Parasitol. Parasites Wildl..

[7] Van Hemert C., Meixell B.W., Smith M.M., Handel C.M. (2019). Prevalence and diversity of avian blood parasites in a resident northern passerine. Parasites Vectors.

[8] Gonzalez-Quevedo C., Pabón A., Rivera-Gutierrez H.F. (2016). Prevalence of haemosporidians in a Neotropical endemic bird area. Avian Conserv. Ecol..

[9] Morel A.P., Webster A., Prusch F., Anicet M., Marsicano G., Trainini G., Stocker J., Giani D., Bandarra P.M., da Rocha M.I.S. (2021). Molecular detection and phylogenetic relationship of Haemosporida parasites in free-ranging wild raptors from Brazil. Vet. Parasitol. Reg. Stud. Rep..

[10] Schmid S., Fachet K., Dinkel A., Mackenstedt U., Woog F. (2017). Carrion crows (*Corvus corone*) of southwest Germany: Important hosts for haemosporidian parasites. Malar. J..

[11] Shurulinkov P., Spasov L., Stoyanov G., Chakarov N. (2018). Blood parasite infections in a wild population of ravens (*Corvus corax*) in Bulgaria. Malar. J..

[12] Schumm Y.R., Wecker C., Marek C., Wassmuth M., Bentele A., Willems H., Reiner G., Quillfeldt P. (2019). Blood parasites in Passeriformes in central Germany: Prevalence and lineage diversity of Haemosporida (*Haemoproteus*, *Plasmodium* and *Leucocytozoon*) in six common songbirds. PeerJ.

[13] Nourani L., Djadid N.D., Rabiee K., Mezerji M.S., Shakiba M., Bakhshi H., Shokrollahi B., Farahani R.K. (2020). Detection of haemosporidian parasites in wild and domestic birds in northern and central provinces of Iran: Introduction of new lineages and hosts. Int. J. Parasitol. Parasites Wildl..

[14] Hanel J., Doležalová J., Stehlíková Š., Modrý D., Chudoba J., Synek P., Votýpka J. (2016). Blood parasites in northern goshawk (*Accipiter gentilis*) with an emphasis to *Leucocytozoon toddi*. Parasitol. Res..

[15] Krone O., Waldenström J., Valkiūnas G., Lessow O., Müller K., Iezhova T.A., Fickel J., Bensch S. (2008). Haemosporidian Blood Parasites in European Birds of Prey and Owls. J. Parasitol..

[16] Lee S.-H., Kwak D., Kim K.-T. (2018). The first clinical cases of Haemoproteus infection in a snowy owl (*Bubo scandiacus*) and a goshawk (*Accipiter gentilis*) at a zoo in the Republic of Korea. J. Vet. Med. Sci..

[17] Giorgiadis M., Guillot J., Duval L., Landau I., Quintard B. (2020). Haemosporidian parasites from captive Strigiformes in France. Parasitol. Res..

[18] Attaran H., Luo J., Bo W., Nabavi R., He H.X. (2021). Haemosporidian Blood Parasites in nestling birds of prey in Mongolia. bioRxiv.

[19] Gao K., Zhou B., Yang L.-X., Dong L., Huang X., Deng W.-H. (2021). How Does Circadian Rhythm Shape Host-Parasite Associations? A Comparative Study on Infection Patterns in Diurnal and Nocturnal Raptors. Diversity.

[20] Baker K.C., Rettenmund C.L., Sander S.J., Rivas A.E., Green K.C., Mangus L., Bronson E. (2018). Clinical effect of hemoparasite infections in snowy owls (*Bubo scandiacus*). J. Zoo Wildl. Med..

[21] Leppert L.L., Dufty A.M., Stock S., Oleyar M.D., Kaltenecker G.S. (2008). Survey of Blood Parasites in Two Forest Owls, Northern Saw-whet Owls and Flammulated Owls, of Western North America. J. Wildl. Dis..

[22] Valkiunas G. (2005). Avian Malaria Parasites and Other Haemosporida.

[23] Chakarov N., Veiga J., Ruiz-Arrondo I., Valera F. (2021). Atypical behavior of a black fly species connects cavity-nesting birds with generalist blood parasites in an arid area of Spain. Parasites Vectors.

[24] Maiorano L., Amori G., Capula M., Falcucci A., Masi M., Montemaggiori A., Pottier J., Psomas A., Rondinini C., Russo D. (2013). Threats from Climate Change to Terrestrial Vertebrate Hotspots in Europe. PLoS ONE.

[25] van Wijk R.E., Bauer S., Schaub M. (2016). Repeatability of individual migration routes, wintering sites, and timing in a long-distance migrant bird. Ecol. Evol..

[26] (2010). Directive 2010/63/EU of the European Parliament and of the Council of 22 September 2010 on the Protection of Animals Used for Scientific Purposes.

[27] Brubaker J.L., Karouna-Renier N.K., Chen Y., Jenko K., Sprague D.T., Henry P.F.P. (2011). A noninvasive, direct real-time PCR method for sex determination in multiple avian species. Mol. Ecol. Resour..

[28] Clements J., Sanchez J.N. (2015). Creation and validation of a novel body condition scoring method for the magellanic penguin (*Spheniscus magellanicus*) in the zoo setting. Zoo Biol..

[29] Samour J. (2015). Avian Medicine.

[30] Fudge A.M., Joseph V. (2000). Disorders of avian leukocytes. Laboratory Medicine: Avian and Exotic Pets.

[31] Tostes R., Dias R.J.P., Martinele I., Senra M.V.X., D’Agosto M., Massard C.L. (2017). Multidisciplinary re-description of *Plasmodium* (*Novyella*) paranucleophilum in Brazilian wild birds of the Atlantic Forest kept in captivity. Parasitol. Res..

[32] Muñoz E., Ferrer D., Molina R., Adlard R.D. (1999). Prevalence of haematozoa in birds of prey in Catalonia, north-east Spain. Vet. Rec..

[33] Tomé R., Santos N., Cardia P., Ferrand N., Korpimaki E. (2005). Factors affecting the prevalence of blood parasites of Little Owls Athene noctua in southern Portugal. Ornis Fenn..

[34] Krone O., Priemer J., Streich J., Sommer P., Langgemach T., Lessow O. (2001). Haemosporida of birds of prey and owls from Germany. Acta Protozool..

[35] Carlson M.L., Proudfoot G.A., Gentile K., Dispoto J., Weckstein J.D. (2018). Haemosporidian prevalence in northern saw-whet owls *Aegolius acadicus* is predicted by host age and average annual temperature at breeding grounds. J. Avian Biol..

[36] Barino G.T.M., Rossi M.F., De Oliveira L., Junior J.L.R., D’Agosto M., Dias R.J.P. (2021). *Haemoproteus syrnii* (Haemosporida: Haemoproteidae) in owls from Brazil: Morphological and molecular characterization, potential cryptic species, and exo-erythrocytic stages. Parasitol. Res..

[37] Ilgūnas M., Himmel T., Harl J., Dagys M., Valkiūnas G., Weissenböck H. (2022). Exo-Erythrocytic Development of Avian Haemosporidian Parasites in European Owls. Animals.

[38] Pornpanom P., Chagas C.R.F., Lertwatcharasarakul P., Kasorndorkbua C., Valkiūnas G., Salakij C. (2019). Molecular prevalence and phylogenetic relationship of *Haemoproteus* and *Plasmodium* parasites of owls in Thailand: Data from a rehabilitation centre. Int. J. Parasitol. Parasites Wildl..

[39] Levin I., Parker G. (2011). Haemosporidian parasites: Impacts on avian hosts. Fowler’s Zoo and Wild Animal Medicine Current Therapy.

[40] Magri A., Galuppi R., Fioravanti M. (2021). Autochthonous *Trypanosoma* spp. in European Mammals: A Brief Journey amongst the Neglected Trypanosomes. Pathogens.

[41] Valkiūnas G., Bairlein F., Iezhova T.A., Dolnik O.V. (2004). Factors affecting the relapse of Haemoproteus belopolskyi infections and the parasitaemia of *Trypanosoma* spp. in a naturally infected European songbird, the blackcap, *Sylvia atricapilla*. Parasitol. Res..

[42] Svobodová M., Weidinger K., Peske L., Volf P., Votýpka J., Vorisek P. (2015). Trypanosomes and haemosporidia in the buzzard (*Buteo buteo*) and sparrowhawk (*Accipiter nisus*): Factors affecting the prevalence of parasites. Parasitol. Res..

[43] Pérez-Rodríguez A., de la Hera I., Bensch S., Pérez-Tris J. (2015). Evolution of seasonal transmission patterns in avian blood-borne parasites. Int. J. Parasitol..

[44] Chagas C.R.F., Valkiūnas G., Guimarães L.D.O., Monteiro E.F., Guida F.J.V., Simões R.F., Rodrigues P.T., Luna E.J.d.A., Kirchgatter K. (2017). Diversity and distribution of avian malaria and related haemosporidian parasites in captive birds from a Brazilian megalopolis. Malar. J..

[45] Hisada Y., Saito K., Asakawa M. (2004). Epidemiological Survey of *Haemoproteus* sp. Found Blakiston’s Owls (*Ketupa blakistoni blakistoni*) on Hokkaido Island, Japan. Jpn. J. Zoo Wildl. Med..

[46] Karadjian G., Puech M.-P., Duval L., Chavatte J.-M., Snounou G., Landau I. (2013). *Haemoproteus syrnii* in *Strix aluco* from France: Morphology, stages of sporogony in a hippoboscid fly, molecular characterization and discussion on the identification of *Haemoproteus* species. Parasite.

[47] Yoshimoto M., Ozawa K., Kondo H., Echigoya Y., Shibuya H., Sato Y., Sehgal R.N.M. (2021). A fatal case of a captive snowy owl (*Bubo scandiacus*) with *Haemoproteus* infection in Japan. Parasitol. Res..

[48] Niedringhaus K.D., Fenton H.M., Cleveland C.A., Anderson A.N., Schwartz D., Alex C.E., Rogers K.H., Mete A., Yabsley M.J. (2018). Case Series: Virulent hemosporidiosis infections in juvenile great horned owls (*Bubo virginianus*) from Louisiana and California, USA. Vet. Parasitol. Reg. Stud. Rep..

[49] Apanius V., Kirkpatrick C.E. (1988). Preliminary Report of *Haemoproteus tinnunculi* Infection in a Breeding Population of American Kestrels (*Falco sparverius*). J. Wildl. Dis..

[50] Marzal A., Bensch S., Reviriego M.I., Balbontin J., De Lope F. (2008). Effects of malaria double infection in birds: One plus one is not two. J. Evol. Biol..

[51] Evans M., Otter A. (1998). Fatal combined infection with *Haemoproteus noctuae* and *Leucocytozoon ziemanni* in juvenile snowy owls (*Nyctea scandiaca*). Vet. Rec..

[52] A Harasym C. (2008). West Nile virus and hemoparasites in captive snowy owls (*Bubo scandiacus*)—Management strategies to optimize survival. Can. Vet. J..

[53] Ziman M., Colagross-Schouten A., Griffey S., Stedman B. (2004). *Haemoproteus* spp. and *Leukocytozoon* spp. in a Captive Raptor Population. J. Wildl. Dis..

[54] Davis A.K., Maney D.L., Maerz J.C. (2008). The use of leukocyte profiles to measure stress in vertebrates: A review for ecologists. Funct. Ecol..

[55] Samour J.H., D’Aloia M.A., Howlett J.C. (1996). Normal haematology of captive saker falcons (*Falco cherrug*). Comp. Haematol. Int..

[56] Wiegmann A., Springer A., Rinaud T., Ottensmann M., Legler M., Krüger O., Fehr M., Chakarov N., Strube C. (2021). The prevalence of *Leucocytozoon* spp. in nestlings of three wild raptor species including implications on haematological and blood chemistry values. Int. J. Parasitol. Parasites Wildl..

[57] Clark N.J., Wells K., Dimitrov D., Clegg S.M. (2016). Co-infections and environmental conditions drive the distributions of blood parasites in wild birds. J. Anim. Ecol..

