# Supplementary material for: A Four-Year Survey of Hemoparasites from Nocturnal Raptors (Strigiformes) Confirms a Relation between Leucocytozoon and Low Hematocrit and Body Condition Scores of Parasitized Birds
Source: Vet Sci. 2023 Jan 12;10(1):54. doi: 10.3390/vetsci10010054 (PMC9865734; doi:10.3390/vetsci10010054)
Supplement: Supplementary file 1 [file vetsci-10-00054-s001.zip › vetsci-2131327-supplementary.pdf]

Supplementary Table S1. Details about distribution of the study population regarding the species, age, gender and body condition score of animals, and the year of sampling.

	**N total**	**Age**		**Gender**			**Body Condition Score**					**Year of sampling**			
		**Young**	**Adult**	**Female**	**Male**	**Unknown**	**0/5**	**1/5**	**2/5**	**3/5**	**4/5**	**2018**	**2019**	**2020**	**2021**
Barn owl	90	72/90	18/90	20/90	20/90	50/90	0/90	0/90	19/90	71/90	0/90	11/90	28/90	28/90	23/90
(*Tyto alba*)		80%	20%	22.2%	22.2%	55.6%	0%	0%	21.1%	78.9%	0%	12.2%	31.1%	31.1%	25.6%
Eurasian eagle-owl	19	3/19	16/19	7/19	5/19	7/19	4/19	2/19	10/19	3/19	0/19	9/19	4/19	3/19	3/19
(*Bubo bubo*)		15.8%	84.2%	36.8%	26.4%	36.8%	21.1%	10.5%	52.6%	15.8%	0%	47.4%	21.1%	15.8%	15.8%
Tawny owl	10	1/10	9/10	1/10	1/10	8/10	1/10	0/10	2/10	7/10	0/10	5/10	1/10	2/10	2/10
(*Strix aluco*)		10%	90%	10%	10%	80%	10%	0%	20%	70%	0%	50%	10%	10%	10%
European owl	10	5/10	5/10	2/10	1/10	7/10	0/10	1/10	1/10	7/10	1/10	8/10	0/10	0/10	2/10
(*Athene noctua*)		50%	50%	20%	10%	70%	0%	10%	10%	70%	10%	80%	0%	0%	20%
Eurasian Scops-owl	4	3/4	1/4	0/4	0/4	4/4	0/4	0/4	0/4	4/4	0/4	3/4	1/4	0/4	0/4
(*Otus scops*)		75%	25%	0%	0%	100%	0%	0%	0%	100%	0%	75%	25%	0%	0%
Northern long-eared owl	1	0/1	1/1	0/1	1/1	0/1	0/1	0/1	1/1	0/1	0/1	0/1	0/1	1/1	0/1
(*Asio otus*)		0%	100%	0%	100%	0%	0%	0%	100%	0%	0%	0%	0%	100%	0%
**TOTAL**	**134**	**84/134**	**50/134**	**30/134**	**28/134**	**76/134**	**5/134**	**3/134**	**33/134**	**92/134**	**1/134**	**36/134**	**34/134**	**34/134**	**30/134**
		**62.7%**	**37.3%**	**22.4%**	**20.9%**	**56.7%**	**3.7%**	**2.2%**	**24.6%**	**68.7%**	**0.7%**	**26.9%**	**25.4%**	**25.4%**	**22.3%**

N = number of raptors; % = percentage
